# Type I Photosensitizers Based on Aggregation-Induced Emission: A Rising Star in Photodynamic Therapy

**DOI:** 10.3390/bios12090722

**Published:** 2022-09-04

**Authors:** Danxia Li, Peiying Liu, Yonghong Tan, Zhijun Zhang, Miaomiao Kang, Dong Wang, Ben Zhong Tang

**Affiliations:** 1 Center for AIE Research, Shenzhen Key Laboratory of Polymer Science and Technology, Guangdong Research Center for Interfacial Engineering of Functional Materials, College of Materials Science and Engineering, Shenzhen University, Shenzhen 518060, China; 2 School of Science and Engineering, Shenzhen Institute of Aggregate Science and Technology, The Chinese University of Hong Kong, Shenzhen 518172, China

**Keywords:** aggregation-induced emission, type I photosensitizers, phototheranostics

## Abstract

Photodynamic therapy (PDT), emerging as a minimally invasive therapeutic modality with precise controllability and high spatiotemporal accuracy, has earned significant advancements in the field of cancer and other non-cancerous diseases treatment. Thereinto, type I PDT represents an irreplaceable and meritorious part in contributing to these delightful achievements since its distinctive hypoxia tolerance can perfectly compensate for the high oxygen-dependent type II PDT, particularly in hypoxic tissues. Regarding the diverse type I photosensitizers (PSs) that light up type I PDT, aggregation-induced emission (AIE)-active type I PSs are currently arousing great research interest owing to their distinguished AIE and aggregation-induced generation of reactive oxygen species (AIE-ROS) features. In this review, we offer a comprehensive overview of the cutting-edge advances of novel AIE-active type I PSs by delineating the photophysical and photochemical mechanisms of the type I pathway, summarizing the current molecular design strategies for promoting the type I process, and showcasing current bioapplications, in succession. Notably, the strategies to construct highly efficient type I AIE PSs were elucidated in detail from the two aspects of introducing high electron affinity groups, and enhancing intramolecular charge transfer (ICT) intensity. Lastly, we present a brief conclusion, and a discussion on the current limitations and proposed opportunities.

## 1. Introduction

Photodynamic therapy (PDT) was first defined in the middle of the 20th century when R. Lipson and S. Schwartz discovered the cancer diagnostic and therapeutic effects of a hematoporphyrin derivative (HpD) [1]. Since then, exploration in the field of PDT has continued without cessation [2,3]. Possessing the distinguished merits of non-invasiveness, high spatiotemporal precision, accurate controllability, and low systemic toxicity, PDT is currently captivating an unprecedented level of research interest as a pioneering and intriguing therapeutic modality, with significant advancements in PDT witnessed in the areas of cancer and various non-oncological disease therapies [4,5]. For example, the effectiveness of PDT in the treatment of skin, neck and superficial bladder cancers [6], as well as pathogen-[7,8] (e.g., propionibacterium acnes [9], human papilloma virus [10,11]) caused infectious diseases, has been experimentally or clinically validated.

Basically, PDT mainly relies on the oxidative reactive oxygen species (ROS), including singlet oxygen (^1^O_2_), superoxide radical (O_2_^•−^), hydroxyl radical (OH^•^), hydrogen peroxide (H_2_O_2_), etc., to play the therapeutic role, as ROS not only can cause direct killing of cancer cells or pathogens via destruction of the cellular components, but can also induce vascular damage as well as acute local and systemic immune response, to jointly eliminate tumors [12]. In the PDT process, ROS are generally produced via two types of photo-triggered reactions (namely, type I and type II) between the photosensitizers (PSs) and surrounding substrates [13]. To be specific, the type I reaction follows hydrogen atom abstraction or electron transfer manner, subsequently leading to the formation of radicals and H_2_O_2_; alternatively, energy transfer from the electronically excited triplet-state PSs to the ground-state molecular oxygen is involved in the type II process accompanying ^1^O_2_ production [14]. In theory, these two competing photoreaction pathways can occur in parallel, but the type of PSs, oxygen concentration, as well as adjacent substrates, always cause one pathway to be dominant in the practical PDT process [15]. Owing to the relatively low excited energy required to form ^1^O_2_ from molecular oxygen, most of the reported PSs are inclined to undergo the oxygen-dependent type II PDT process, the therapeutic efficacy of which, however, is fatally impaired by the untoward predicament of the hypoxic microenvironments of the pathological tissues, such as the interior of a solid tumor or bacterial infection site [16,17]. By contrast, type I PDT has been proven to hold great potential in breaking through this inherent bottleneck, since diminished oxygen supply is required in a type I reaction [18].

The distinctive hypoxia tolerance and favorable therapeutic performance of type I PDT in hypoxic pathological tissues have largely spawned the evolution of type I PSs over the past several decades [19]. To date, profiting from the enormous efforts of people dedicated to the pursuit of puissant type I PDT, multifarious materials have been developed as type I PSs, including but not limited to metal oxides [20] (e.g., TiO_2_), carbon-based nanomaterials [21,22] (e.g., carbon dots, *g*-C_3_N_4_), organic–inorganic hybrids [23,24] (e.g., metal–organic framework), transition metal complexes [25] (e.g., Ru(II) complexes), and organic molecules [26,27]. Unlike inorganic materials which suffer from poor biodegradability, complicated pharmacokinetics and worrisome biosecurity, organic molecules with their distinct advantages of favorable biocompatibility, satisfactory metabolism, facile processability, excellent reproducibility, structural diversity and easy tunability, stand out as a promising option for practical bioapplications [28,29]. In particular, some organic PSs are capable of enabling fluorescence imaging (FLI)-guided PDT due to their intrinsic fluorescence emission feature, which represents an important category of photo-driven theranostics [30,31,32].

The most eye-catching representatives are PSs with aggregation-induced emission (AIE) characteristics [33]. In addition to the common advantages of organic PSs, as a class of novel organic fluorophores, AIE PSs uniquely exhibit incomparable attributes of aggregation-induced emission and aggregation-induced generation of ROS (AIG-ROS), due to their twisted structures as well as their ornamentation with rich rotators or vibrators [34]. To be specific, AIE PSs usually show faint fluorescence emission and ROS generation in the dissolved state, because of the intramolecular motion-resulted excited energy consumption [35]. This nonradiative thermal dissipation, however, can be effectively suppressed upon aggregation due to the restriction of intramolecular motion (RIM), which consequently promotes fluorescence as well as the ROS generation-involved intersystem crossing (ISC) channel at the aggregate state [36]. In addition, the twisted conformation of AIE PSs can also significantly weaken the intermolecular π-π stacking after aggregation and, thus, ultimately contribute to AIE and AIG-ROS features [37]. Since most organic PSs are structurally hydrophobic and inevitably tend to form aggregates in aqueous physiological environments, by taking full advantage of aggregation, AIE PSs have earned a wider scope of application in the biomedical field, in contrast with traditional aggregation-caused quenching (ACQ) PSs, which usually suffer from diminished fluorescence emission and decreased ROS production at the aggregate state because of their rigidly planar conformations-caused competitive energy consumption [38].

By virtue of these unique superiorities of AIE luminogens (AIEgens) in serving as ideal PSs, an increasing number of AIE-active type I PSs have been rationally designed, and the resultant high-efficiency type I PDT has been successfully achieved in various application scenarios in recent years [39,40]. In this review, we will attempt to summarize the recent advancements of type I AIE PSs, emphasizing their molecular design strategies and rationales. First, as a theoretical basis, the basic photophysical and photochemical mechanism of the type I reaction is introduced. Based on this, current design strategies to obtain high-performance type I AIE PSs are highlighted and elaborated in detail from two aspects: introducing functional groups with high electron affinity, and enhancing intramolecular charge transfer (ICT) intensity. Then, the wide bioapplications of type I AIE PSs in the photodynamic eradication of tumors and pathogen-caused infectious diseases, as well as inhibition of harmful algae bloom, are separately showcased. Last, the current limitations, challenges, and perspectives for the future development of type I AIE PSs are discussed.

## 2. Basic Principles of Type I PDT

**Figure 1:**
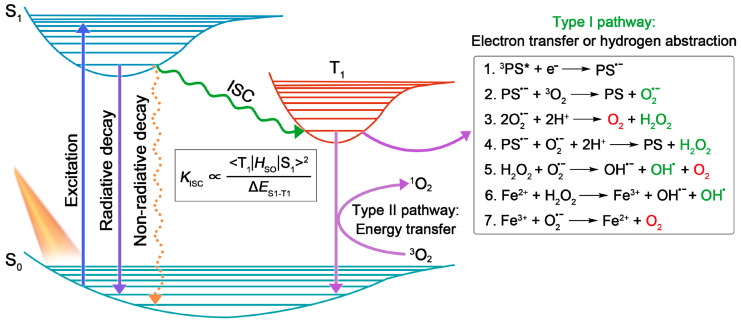
The illustration shows the working mechanisms of PSs described with the Jablonski diagram. The inserted box in the middle shows the ISC rate equation. The inserted box on the right shows the related cascaded reactions during the type I process.

In general, PSs, light source, and substrates, are recognized as three essential elements in PDT [4]. According to the Jablonski diagram shown in Figure 1, the PSs at ground singlet state (S_0_) will, firstly, be excited to the unstable excited singlet state (S_1_) upon light irradiation, and then return to their ground state via radiative or non-radiative decay in the manner of fluorescence emission or heat production. Notably, provided that the energy gap between S_1_ and T_1_ (Δ*E*_S1–T1_) is small enough, the excited singlet state PSs can preferably reach the relatively stable excited triplet-state (T_1_) by undergoing the ISC process, in which state the PSs can survive long enough to carry out different photochemical reactions (including type I and type II) with surrounding substrates, to yield ROS [6]. Here, type I process will be emphasized.

Unlike the type II reaction involving a direct energy transfer to the triplet-state molecular oxygen to produce ^1^O_2_, the type I process refers to the production of H_2_O_2_ and radicals (e.g., O_2_^•−^, OH^•^) via several cascade electron transfer and hydrogen atom abstraction procedures [1]. Specifically speaking, the type I pathway usually begins with an initial one-electron reduction of the triplet-state PS (^3^PS*) with the production of a PS radical anion (PS^•−^) (Reaction 1), which can further transfer one electron to molecular oxygen to produce O_2_^•−^ (Reaction 2). By virtue of the disproportionation catalyzed by superoxide dismutase (SOD) (Reaction 3) or another one-electron reduction by PS^•−^ (Reaction 4), O_2_^•−^ can be reduced to H_2_O_2_. Then, the generated H_2_O_2_ can ultimately be transformed into highly oxidative OH^•^ by reacting with O_2_^•−^ or Fe^2+^, known as the Haber–Weiss reaction (Reaction 5) and Fenton reaction (Reaction 6), respectively [41]. In this respect, the Fenton reaction can be augmented since the Fe^3+^ produced in this process can be reduced to Fe^2+^ by O_2_^•−^ for recycling (Reaction 7) [42].

By making full utilization of the disproportionation reaction, Haber–Weiss reaction, or Fenton reaction, type I PDT has been proven to exhibit superior therapeutic outcomes in hypoxic environments, in contrast with type II PDT. This can be explained from the following aspects: (1) O_2_^•−^ (few seconds) is recognized as having a much longer half-life than ^1^O_2_ (10^−5^ s), which endows O_2_^•−^ with a relatively long diffusion distance compared with others [43,44]; (2) featured with robust oxidative characteristic, OH^•^ is the most biologically aggressive reactive oxygen centered radical that can cause direct damage to various vital biomacromolecules, thus, exerting amplified PDT response [45,46,47]; (3) unlike the heavy O_2_ consumption of the type II pathway, the O_2_ needed in the type I pathway can be recycled among its reactions, which can enable the limited O_2_ in hypoxic conditions to be fully utilized, and endow type I PDT with good hypoxia tolerance [18].

## 3. Design Strategies of Type I AIE PSs

Encouraged by the distinguished merits of AIE and AIG-ROS of AIE PSs, as well as the great potential that type I PSs hold in practical applications, type I AIE PSs have emerged with abundance in the last five years. As depicted in Figure 1, the ISC process is the foremost step that bears the brunt of following ROS generation, thus, a high ISC rate (*k*_ISC_) ensuring ample excited triplet-state production is considered as a precondition to boost the photosensitizing properties of PSs. To date, a large number of studies have emerged demonstrating how to promote the ISC process of AIE PSs on the basis of the ISC rate equation (Figure 1), such as a donor (D)–acceptor (A) structured molecular engineering strategy for minimized Δ*E*_S1–T1_, and spin–orbit coupling (SOC) enhancement strategy for improved SOC, etc. [30]. However, regarding specifically promoting the generation of type I free radical species rather than type II, authoritative and specific strategies are currently still relatively deficient, although several examples of type I AIE PSs have been provided. According to the basic principles of electron transfer in the type I pathway, herein we summarize and classify the currently reported approaches of building type I AIE PSs into two strategies: introducing functional groups with high electron affinity, and enhancing ICT intensity.

### 3.1. Introducing High Electron Affinity Groups

**Figure 2:**
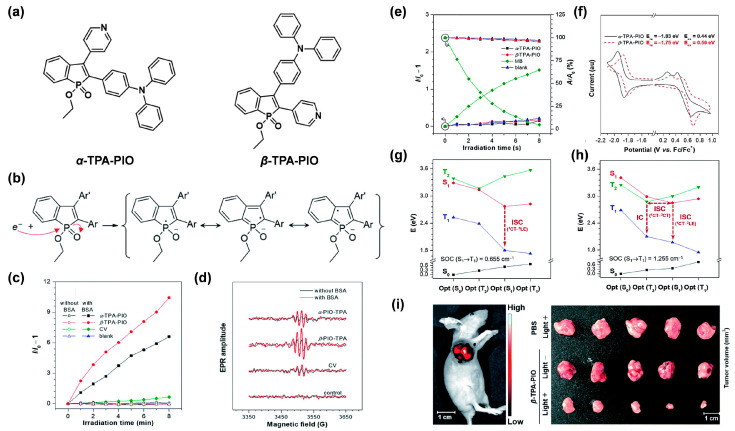
(**a**) The chemical structures of *α*-TPA-PIO and *β*-TPA-PIO. (**b**) Resonance structures of the PIO radical ions. (**c**) Relative fluorescence intensity of hydroxyphenyl fluorescein (HPF) for OH^•^ detection. (**d**) Electron spin resonance (ESR) signals of 5-tert-butoxycarbonyl-5-methyl-1-pyrroline-N-oxide (BMPO) for free radical ROS detection of *α*-TPA-PIO and *β*-TPA-PIO with or without bovine serum albumin (BSA). (**e**) Relative fluorescence intensity of singlet oxygen sensor green (SOSG) and decomposition rates of 9,10-anthracenediyl-bis(methylene) dimalonic acid (ABDA) for ^1^O_2_ detection of *α*-TPA-PIO, *β*-TPA-PIO and MB. (**f**) Cyclic voltammograms of *α*-TPA-PIO and *β*-TPA-PIO. SOC value and ISC process of (**g**) *α*-TPA-PIO, and (**h**) *β*-TPA-PIO. (**i**) Images of mouse and tumors at 24 h post-injection of *β*-TPA-PIO. Reprinted with permission from [48], copyright 2020, Royal Society of Chemistry.

After gaining insight into the mechanism of the type I process, it can easily be observed that before transferring the electron to O_2_ to yield O_2_^•−^, ^3^PS* first needs to capture one electron from its surroundings. Thus, employing functional groups with high electron affinity will contribute to this step, since they can serve as electronic transfer intermediates. For example, Zhao et al. [48] designed and synthesized two isomeric type I AIE PSs, named *α*-TPA-PIO and *β*-TPA-PIO, by employing phosphindole oxide (PIO) as an electron acceptor, and triphenylamine (TPA) as an electron donor (Figure 2a). It was proven that owing to the high electron affinity of the PIO core, both of the triplet *α*-TPA-PIO and *β*-TPA-PIO could attract one electron from the surrounding substrates and accordingly convert to the intermediate radical anions, *α*-TPA-PIO^•−^ and *β*-TPA-PIO^•−^, respectively, which could be stabilized by several resonance structures (Figure 2b). As expected, *α*-TPA-PIO and *β*-TPA-PIO were demonstrated to exhibit admirable free radical but low ^1^O_2_ generation efficiency (Figure 2c–e), evidently suggesting that the introduction of high electron-affinity PIO indeed favors type I ROS generation by stabilizing an external electron to form radical anion intermediates. Notably, *β*-TPA-PIO, which possessed stronger electron-accepting ability as indicated by its lower reduction potential, exhibited higher type I ROS generation efficacy than *α*-TPA-PIO (Figure 2f), thus, confirming the superiority of strong electron affinity in dominating the type I pathway. In addition, the higher type I ROS generation efficiency of *β*-TPA-PIO also gave the credit to the larger SOC value, as well as the multichannel ISC pathways revealed by quantum mechanical calculation (Figure 2g,h). By virtue of the good targeting ability to endoplasmic reticulum (ER) of cells, *β*-TPA-PIO could efficiently induce ER-stress-mediated apoptosis and autophagy via type I PDT, even in a hypoxic environment. Moreover, distinct in vivo fluorescence imaging of the tumor, and remarkable tumor ablation, could successfully be achieved under light irradiation (Figure 2i). Therefore, this work presented a feasible protocol for type I AIE PSs by introducing high electron-affinity groups to capture and stabilize the external electron, which provided the photochemical basis of type I PDT.

### 3.2. Enhancing ICT Intensity

#### 3.2.1. Donor Engineering Based on Anion-π^+^ AIE Systems

As mentioned above, the type I pathway usually starts at the one-electron transfer step from adjacent substrates to ^3^PS^*^. Therefore, it can be speculated that providing electron-rich environments is beneficial for ^3^PS^*^ to capture the external electron and undergo the type I process upon light irradiation. This assumption has been substantiated by Ding et al. [49]. They successfully modulated the photoreaction of a type II PS from the type II to the type I pathway by encapsulating the PS using electron-rich poly(ethylene glycol)-*b*-poly(2-(diisopropylamino)ethyl methacrylate (PEG-*b*-PDPA) as the coating substrate. Motivated by this work, a series of anion-π^+^ AIE PSs were successively constructed in the pursuit of efficient type I ROS generation, among which anion groups were introduced to offer electron-rich environments [50,51].

**Figure 3:**
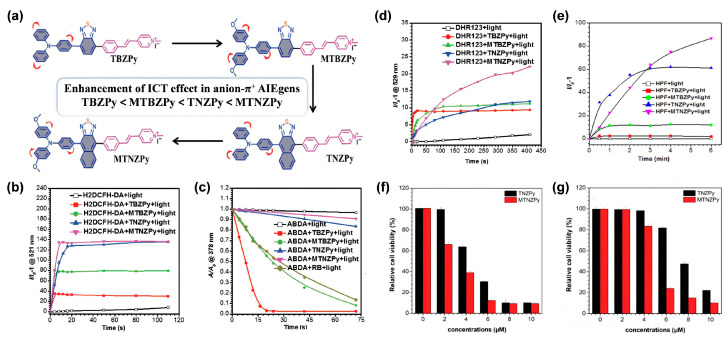
(**a**) Chemical structures and the order of ICT effect of TBZPy, MTBZPy, TNZPy, and MTNZPy. (**b**) 2,7-dichlorodihydrofluorescein diacetate (DCFH-DA), (**c**) ABDA, (**d**) Dihydrorhodamine 123 (DHR 123) and (**e**) HPF for total ROS, ^1^O_2_, O_2_^•−^, OH^•^ detection, respectively. The survival rate of HeLa Cells for a range of concentrations of TNZPy and MTNZPy in (**f**) normoxic environments and (**g**) hypoxic environments under light irradiation. Reprinted with permission from [50], copyright 2020, Wiley-VCH.

In view of the photomechanical basis of the type I process, strong ICT intensity of PSs was also conjectured to be helpful in donating to the type I pathway under the condition of an electronic-rich environment, since enhanced ICT could facilitate the ISC process, thus, promoting ROS generation capacity. In this regard, studies have revealed that anion-π^+^ PSs fabricated with a strong ICT characteristic would produce type I ROS more efficiently. For instance, through enhancing the ICT strength of the anion-π^+^ system, Wang et al. [50] successfully obtained robust type I AIE PSs. They firstly synthesized a novel series of anion-π^+^ structured AIE PSs (TBZPy, MTBZPy, TNZPy, MTNZPy) with enhanced electron-donating ability. As Figure 3a illustrates, TPA and its methoxy-substituted derivative (MTPA), as well as electron-rich heteroatoms (S, N) containing benzo-2,1,3-thiadiozole (BZ)/naphtho[2,3-c][1,2,5]thiadiazole (NZ) moieties groups, served as collaborative AIE-active donors, and the styrylpyridine cations worked as electron acceptors to ensure the ICT intensity; simultaneously, the iodide anion and collaborative donors with strong reducibility were in charge of providing an electron-rich environment to ^3^PS*. Due to the gradually enhanced electron-donating ability from TBZPy to MTNZPy, MTNZPy possessed the strongest ICT intensity, in the order of TBZPy < MTBZPy < TNZPy < MTNZPy, which was manifested by their absorption spectra in different solvents. It was demonstrated that the generation of ^1^O_2_ species gradually decreased, in line with the enhancement of ICT strength, while both total ROS and type I ROS generation efficiency of AIE PSs matched the trend of ICT strength (Figure 3b–e), suggesting the pivotal role of strong ICT strength in facilitating type I ROS species generation. Further experiments revealed that the obtained type I AIE PSs (TNZPy, MTNZPy) could target mitochondria and lysosomes simultaneously, and exhibited low dark toxicity and admirable PDT therapeutic efficiency for HeLa cells in both normoxic and hypoxic conditions (Figure 3f,g), attributing to the highly efficient type I ROS production inside cells. Additionally, their good performances in FLI-guided PDT were subsequently demonstrated in in vivo tumor models. Similarly, the feasibility of this strategy was also confirmed by Zhu and coworkers [51]. In their work, type I ROS generation was significantly boosted by enhancing the electron-donating ability to promote the ICT intensity of electron-rich anion-π^+^ AIEgens.

#### 3.2.2. Acceptor Planarization

**Figure 4:**
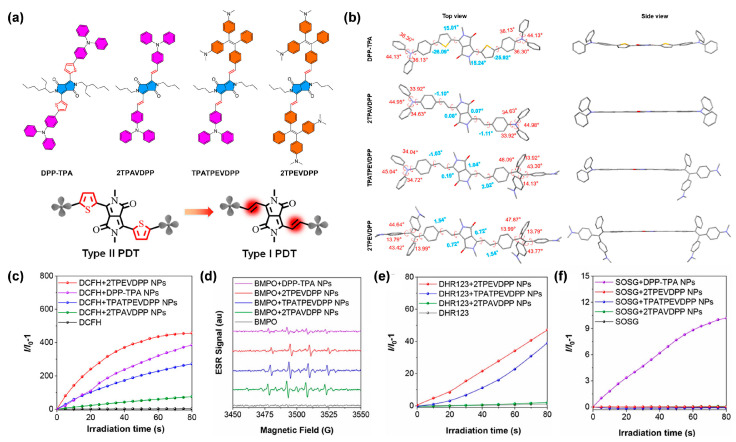
(**a**) Chemical structures and design principles of DPP-TPA, 2TPAVDPP, TPATPEVDPP and 2TPEVDPP. (**b**) Optimized conformation of DPP-TPA, 2TPAVDPP, TPATPEVDPP and 2TPEVDPP. The text with blue or red color shows the torsion angles of the molecular backbones. (**c**) Relative fluorescence intensity of DCFH for total ROS detection of DPP-TPA, 2TPAVDPP, TPATPEVDPP and 2TPEVDPP. (**d**) ESR signals of BMPO for free radical ROS detection of DPP-TPA, 2TPAVDPP, TPATPEVDPP and 2TPEVDPP. Relative fluorescence of (**e**) DHR123 for O_2_^•−^ detection, and (**f**) SOSG for ^1^O_2_ detection, of DPP-TPA, 2TPAVDPP, TPATPEVDPP and 2TPEVDPP. Reprinted with permission from [52], copyright 2022, American Chemical Society.

In addition to enhancing the electron-donating ability of donors in anion-π^+^ AIEgens, Wang et al. [52] proposed a parallel strategy of acceptor planarization to enhance the ICT intensity, achieving the transformation from type II PSs to type I PSs. They designed and synthesized three AIE compounds, namely, 2TPAVDPP, TPATPEVDPP and 2TPEVDPP, using a planar core (vinyl-substituted DPP) as the electron acceptor. As a contrast, the previously reported DPP-TPA with thiophene-substituted DPP working as the twisted acceptor core, was also synthesized (Figure 4a). The optimized conformation (Figure 4b) revealed that the dihedral angle between DPP and donor–acceptor linker was around 15° for DPP-TPA, while it decreased to nearly 0° for those three AIE PSs, indicating that the variation of donor–acceptor linker from thienyl (DPP-TPA) to vinyl (2TPAVDPP, TPATPEVDPP and 2TPEVDPP) would bring about better planarity and larger π-conjugation. It was further demonstrated that 2TPAVDPP, TPATPEVDPP and 2TPEVDPP with a planar acceptor exhibited superior type I and inferior type II ROS generation capacity than DPP-TPA, as expected (Figure 4c–f). These results showed that better planarity and larger π-conjugation could effectively promote type I ROS production by enhancing ICT and D−A interaction to facilitate the ISC process, providing an alternative approach for constructing type I AIE PSs.

## 4. Applications of Type I AIE PSs

On the basis of the mechanism described above, type I PSs exhibit relatively low external O_2_ requirements, owing to the recyclable O_2_ utilization in the type I ROS generation process. Intrinsically, type I AIE PSs enable the crafty integration of aggregation-induced fluorescence emission and enhanced ROS generation with minimized O_2_ dependence, presenting significant theranostic potential in different biomedical applications, including, but not limited to cancer ablation, bacterial infection elimination, and harmful algal bloom suppression.

### 4.1. The Anti-Tumor Applications

Due to the aggressive proliferation of cancer cells and insufficient blood supply, hypoxia often takes place in the microenvironments of solid tumors, thus, severely hindering the generation of type II ROS as it is highly dependent on ambient O_2_ concentration. Conversely, type I PDT has manifested great potential in ablating hypoxic tumors, profiting from its lower O_2_ demand nature. Based on this, AIE PSs featuring type I ROS-generating properties will be ideal candidates for potent PDT, with superb therapeutic outcomes.

#### 4.1.1. FLI-Guided Type I PDT

For the purpose of redshifted absorption and emission wavelengths, as well as boosted theranostic performance, AIE PSs and other organic PSs are generally engineered to contain multiple aromatic rings and/or large conjugated units in their molecular structures, giving rise to their high hydrophobicity. In order to facilitate the in vivo biological applications, hydrophobic AIE PSs are commonly encapsulated within nanovehicles based on amphiphilic biocompatible matrices to form well-dispersed AIE nanoparticles (NPs) in aqueous physiological environments [53]. Additionally, bright fluorescence, excellent ROS production and enhanced permeability and retention (EPR) effect-driven tumor location can be successfully achieved, simultaneously, after nanofabrication, since the aggregation of AIE PSs within the intraparticle limited room is capable of effectively astricting their active intramolecular motions, thus, blocking nonradiative thermal dissipation and saving the excited state energy for the fluorescence and ISC pathway [28,31]. In addition to passively targeted tumor enrichment by the EPR effect, actively targeting transport of AIE PSs favored by specific recognition will be able to further enhance PDT efficacy. From those, Lou et al. [54] developed an amphiphilic polymeric matrix with conjugated targeting peptides to co-assemble with a type I AIE PS of TTB to fabricate tumor-specific targeting TTB NPs for amplifying type I photodynamic cancer treatment. In addition, Duo et al. [55] put forward an innovative protocol for efficiently targeted delivery of type I AIE PSs to tumor tissues by taking full advantage of the hypoxia condition in solid tumors and selective hypoxia tropism of some bacteria. For this approach, a novel bacteria-based AIE hybrid system was built, enabling the powerful delivery of type I AIE PS of TBP-2 into the hypoxic tumor microenvironments for hypoxia-tolerant PDT of orthotopic colon cancer.

Considering that the effective killing range of ROS is typically confined to the immediate vicinity of PSs on a subcellular scale, an appropriate organelle-targeting location of PSs is, therefore, highly desired for implementing final PDT outcomes. Different subcellular organelles play their own unique roles in maintaining the normal physiological function of cells. It has been acknowledged that the organelles, including cell membrane, mitochondria, lysosomes, ER, and nucleus, are all valid sites for performing PDT [56]. To date, diverse subcellular organelle-targeted type I AIE PSs-based anti-tumor systems have been exploited in succession [57]. For instance, Feng et al. [58] developed a class of cationic AIE PSs possessing a specific tumor cell mitochondrial targeting feature to facilitate both type I and type II PDT. Zhao et al. [48] reported two type I AIE PSs to obtain selective accumulation in the ER and effectively arouse ER-stress-mediated cell apoptosis and autophagy upon PDT, by producing highly oxidizing type I radicals under light illumination. In addition, Tang et al. [59] proposed a useful molecular design guideline for constructing efficient AIE PSs and tailoring their organelle specificity.

**Figure 5:**
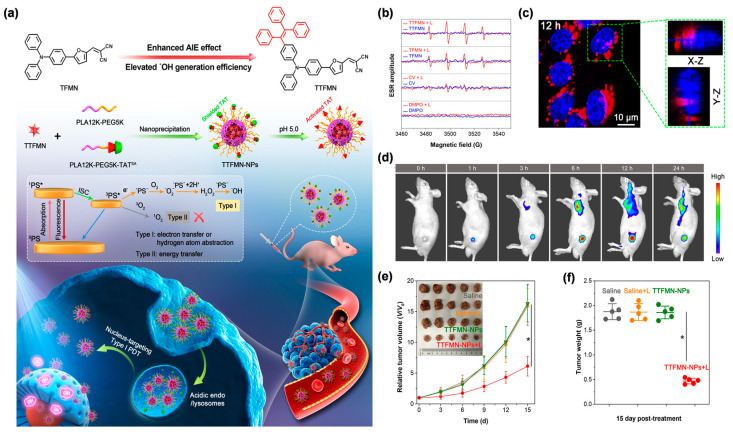
(**a**) Illustration of molecular design principle, nanotheranostics fabrication, and its application in nucleus-targeted type I photodynamic cancer treatment. (**b**) ESR analysis for OH^•^ generation of TTFMN and TFMN after white light irradiation (200 mW/cm^2^). (**c**) CLSM images of nuclear targeting delivery of TTFMN-NPs (2 µg/mL TTFMN) after incubation with 4T1 cells for 12 h. The blue color represents the fluorescence of Hoechst 33342 for locating cell nucleus and the red color represents the fluorescence of TTFMN-NPs. (**d**) Time-dependent in vivo FLI of tumor-bearing mice after injection with TTFMN-NPs. (**e**) The growth curves of tumors in different treatment groups (*n* = 5, * *p* < 0.001). (**f**) The tumor weights of mice after treatments for 15 days (*n* = 5, * *p* < 0.001). Reprinted with permission from [61], copyright 2021, Wiley-VCH.

Among the various subcellular organelles, of particular importance is the cell nucleus as it dominates the cellular gene expression, metabolism and proliferation [60]. Moreover, the DNA and RNA parts of the nuclei are very sensitive to type I ROS due to its extremely high chemical reactivity [61]. In view of this, Wang et al. [61] explored, for the first time, a nucleus-targeting PDT strategy based on type I AIE PSs, by making full use of theranostic agents and nanocarrier systems (Figure 5a). Two AIE PSs, named TFMN and TTFMN, with typical D–A structures and sufficient molecular rotors, were firstly designed and synthesized. Compared with TFMN, TTFMN was equipped with additional TPE moiety in structure, which endowed it with much better AIE peculiarity. Various ROS indicators were employed to distinguish the ROS species produced by TFMN and TTFMN, through fluorescence spectroscopy and ESR measurements, which was discriminated to type I ROS of OH^•^. Moreover, the TTFMN showed stronger ESR signal intensity than TFMN (Figure 5b), indicating its better generation capacity of OH^•^, which was attributed to its superior AIE tendency and smaller Δ*E*_S1–T1_. With the help of a lysosomal acid-activated nuclear localization signal peptide (TAT)-modified amphiphilic polymer, the resultant TTFMN-loaded NPs (TTFMN-NPs) exhibited nucleus-anchoring delivery ability, visualized by the intrinsic fluorescence property of TTFMN (Figure 5c). Further in vivo investigations uncovered that TTFMN-NPs with good biosecurity and long blood circulation time could specifically accumulate at tumor sites (Figure 5d). Upon white light irradiation, TTFMN-NPs induced high-efficiency tumoricidal results with a 75.1% tumor growth inhibition rate (Figure 5e,f). This work offered a new perspective in the construction of type I PS-based and nucleus-targeted nanotheranostic systems.

**Figure 6:**
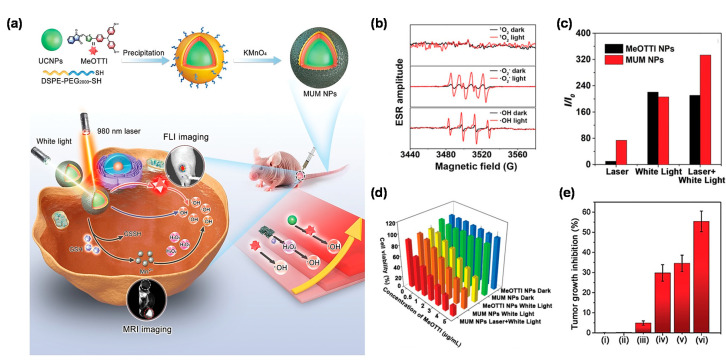
(**a**) Chemical structure of MeOTTI and schematic illustration of triple-jump photodynamic theranostic protocol. (**b**) ROS generation type of MeOTTI determined by ESR test. (**c**) ROS production efficiency of MeOTTI NPs and MUM NPs at the same MeOTTI concentration under the irradiation of different light sources. (**d**) Cell viability of 4T1 cells treated with different conditions. (**e**) Tumor inhibition ratios of mice after different treatments, namely: (i) PBS, (ii) MUM NPs, (iii) 980 nm laser and white light, (iv) MeOTTI NPs and white light, (v) MUM NPs and white light, (vi) MUM NPs, 980 nm laser and white light (*n* = 5, * *p* < 0.001). Reprinted with permission from [63], copyright 2021, Wiley-VCH.

At present, most AIE PSs can only be effectively excited by short wavelength of UV or visible lights. However, the shallow penetration depth of the excited light presented a major scientific challenge for AIE PSs to treat deep-seated tumors. Based on this, the combination of rare earth doped upconversion NPs (UCNPs) would provide an effective solution to this problem, since UCNPs can serve as a near-infrared (NIR) light transducer to harness and convert the NIR laser to UV-visible light, enabling the construction of robust NIR laser excitable nanotheranostic systems [62]. Encouraged by the synergistic effect of combining UCNPs and AIE PSs toward cancer therapy, Wang et al. [63] creatively designed and developed a triple-jump photodynamic nanotheranostic agent, termed MUM NPs, by integrating a type I AIE PS of MeOTTI into the multifunctional nanoplatform built by UCNPs and manganese dioxide (MnO_2_), for enhanced theranostic outputs in PDT (Figure 6a). Specifically, MeOTTI was engineered to afford the type I ROS capacity verified by the ESR test (Figure 6b). With the aid of UCNPs whose emission spectrum matched well with the absorption spectrum of MeOTTI, the resulting Förster resonance energy transfer (FRET) effect between UCNPs and MeOTTI not only achieved the excitation light extension from UV-visible to NIR region, but also significantly elevated the ROS generation efficiency (Figure 6c). Attractively, the introduction of the MnO_2_ component was aimed at depleting the intracellularly upregulated glutathione (GSH), thus, significantly facilitating the production of highly oxidative type I ROS in cells. Meanwhile, the yielded Mn^2+^ was also able to catalyze the intracellular H_2_O_2_ to generate OH^•^, as well as for magnetic resonance imaging (MRI). Therefore, the triple-jump type I ROS generation of MUM NPs could be smoothly achieved inside the tumor cells after NIR laser irradiation. This splendid triple-jump photodynamic theranostic protocol was confirmed by a series of cell and animal experiments (Figure 6d,e).

**Figure 7:**
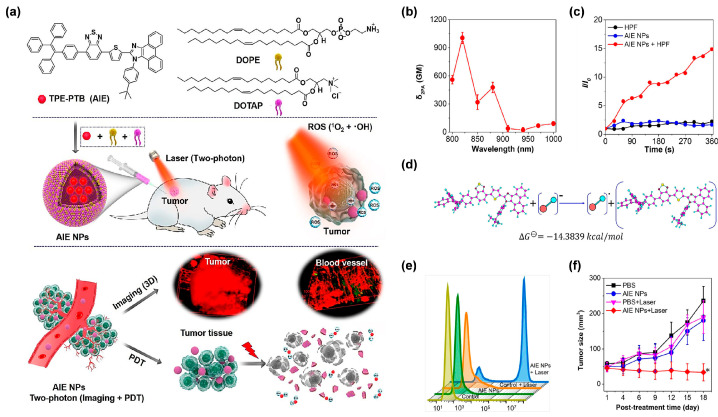
(**a**) Chemical structure of TPE-PTB and illustration of two-photon-excited FLI-guided PDT applications. (**b**) δ_2PA_ of the TPE-PTB NPs under different excitation wavelengths. (**c**) OH^•^ production ability of TPE-PTB NPs indicated by HPF. (**d**) Mechanism and calculation of OH^•^ generation analysis. (**e**) Flow cytometry of A375 cells stained by PI after treatment with different conditions. (**f**) Tumor growth curves of mice in different treatments. (* *p* < 0.05). Reprinted with permission from [66], copyright 2020, American Chemical Society.

In addition to being assisted by UCNPs, exploring AIE PSs with an outstanding two-photon absorption property was another effective method to break through the obstacles encountered by short-wavelength excitation [64]. Moreover, AIE PSs have proved to be promising candidates for developing two-photon excitable PDT agents, as the two-photon absorption cross section (δ_2PA_) of AIE PSs could obviously be raised by simply increasing their loading amount in the NPs, exhibiting a unique aggregation-enhanced nonlinear optical effect [65]. Generally, the wavelength in two-photon excitation is twice as long as that of one-photon absorption, thus, making the NIR light-excitable photodynamic theranostics feasible. In this regard, Tang et al. [66] constructed amphiphilic lipids-enveloping AIE NPs by encapsulating a tactfully designed two-photon excitable type I AIE PS (TPE-PTB) (Figure 7a). With strong D–A interaction and effective π-conjugation strength, TPE-PTB-formed NPs resulted in a high δ_2PA_ of 560 GM under 800 nm two-photon laser irradiation (Figure 7b). Moreover, TPE-PTB NPs exhibited a far-red fluorescence emission with a high quantum yield of 23%. These advantageous superiorities enabled TPE-PTB NPs to image deep-seated tumors and vessels with a high spatial resolution on a mouse melanoma model. Notably, type I ROS species of OH^•^ could be effectively generated by AIE NPs under 800 nm laser illumination (Figure 7c). Further mechanistic explanation showed that TPE-PTB could take one electron from an environmental hydroxyl anion to form anionic PS, for a subsequent series of photochemical reactions, to eventually generate OH^•^ (Figure 7d). As shown in Figure 7e, a live–dead staining experiment showed that TPE-PTB NPs plus NIR laser irradiation could cause more than 90% cell death rate as analyzed by flow cytometry, while no obvious cell death was found in other control groups. More importantly, TPE-PTB NPs performed well for in vivo FLI-guided type I two-photon PDT, with significant inhibition of tumor growth (Figure 7f). In addition, TPE-PTB was able to be effectively cleared from the mouse body after completing the treatment, guaranteeing favorable in vivo biosecurity. A potent NIR-excited type I PDT nanoplatform based on AIEgens was successfully constructed in this work.

#### 4.1.2. The Cooperation of Type I PDT and PTT

**Figure 8:**
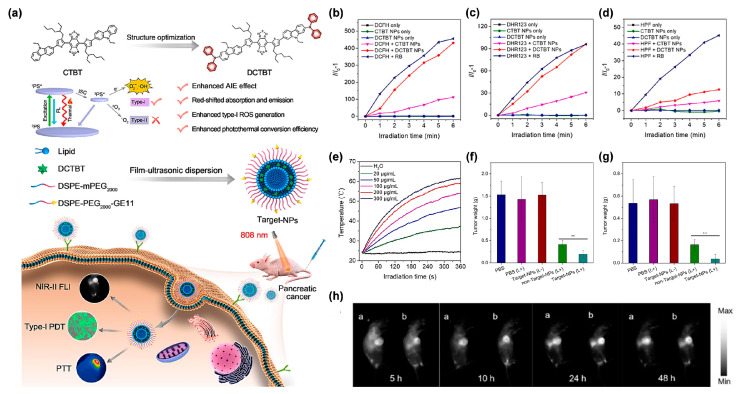
(**a**) Schematic illustration of molecular design and properties, as well as the preparation of target NPs and their application in NIR-II FLI-guided type I PDT-PTT pancreatic cancer therapy. Variation in PL intensity (*I*/*I*_0_) of (**b**) DCFH for total ROS detection, (**c**) DHR123 for O_2_^•−^ detection, and (**d**) HPF for OH^•^ detection. (**e**) Photothermal performance of DCTBT NPs of different concentrations upon laser irradiation (808 nm, 0.8 W/cm^2^, 6 min). (**f**) The average tumor weights of the subcutaneous PANC-1 tumor-bearing mice after different treatments recorded on day 17 (** *p* < 0.01). (**g**) The average tumor weights of the orthotopic PANC-1 tumor-bearing mice after different treatments recorded on day 16 (** *p* < 0.01). (**h**) In vivo NIR-II fluorescence images of subcutaneous PANC-1 tumor-bearing mice at different monitoring times after administration of lip-DCTBT NPs, a: non target NPs, b: target NPs. Reprinted with permission from [67], copyright 2022, Elsevier.

Despite the low O_2_ dependence of the type I process enduing it with hypoxia tolerance, O_2_ is still requisite during type I PDT. Therefore, the efficiency of type I PDT can be further improved by appropriately increasing the O_2_ concentration of the lesions. Recent reports have demonstrated that the cooperation of type I PDT and photothermal therapy (PTT) would be a superb strategy to conquer their respective shortcomings and boost therapeutic outcomes [31,32]. This is because PTT can generate localized heat, not only for cancer ablation, but also to increase the O_2_ supply in the tumor tissues by virtue of raising the blood flow, thus, promoting type I PDT efficiency, which conversely, further improves the treatment outcome of PTT [37]. However, the construction of the type I PDT-PTT combinational system based on a single organic molecule is a challenging task, since the energy dissipation channels are generally competitive. Under this circumstance, AIE molecules have a remarkable capability which can tailor the equilibrium of energy dissipation by regulating the intramolecular motions. In 2022, Wang et al. [67] reported a multifunctional type I AIE PS, namely, DCTBT, applying to a second near-infrared (NIR-II) FLI-guided type I PDT, simultaneous with high-efficiency PTT functions, for pancreatic cancer, the king of cancer [68,69,70]. A conjugated small molecule (CTBT) was modified with diphenylamine moieties on both of the two ends, to endow DCTBT with sufficient intramolecular motions for enhanced PTT efficiency, stronger electron D–A interaction for longer absorption and emission wavelengths, and smaller Δ*E*_S1–T1_ for improved ROS generation ability (Figure 8a). After employing different ROS indicators (Figure 8b–d), DCTBT NPs fabricated by using DSPE-PEG_2000_ as the encapsulation matrix were demonstrated to have distinguished O_2_^•−^ and moderate OH^•^ generation efficiencies. Moreover, DCTBT NPs exhibited comparable O_2_^•−^ generation ability to rose bengal (RB), which was much stronger than that of CTBT NPs. In vitro photothermal experiments (Figure 8e) showed that DCTBT NPs achieved concentration-dependent photothermal performance, and the highest temperature could reach up to 62.8 °C under 808 nm laser irradiation for 5 min as the concentration rose to 300 μg/mL, which implied the excellent photothermal performance of DCTBT NPs. With the aid of the GE11 peptide, a specific ligand for epidermal growth factor receptor (EGFR), target NPs, whose surface was decorated with DSPE-PEG_2000_-GE11, were prepared to facilitate the intracellular intake of lip-DCTBT NPs (the DCTBT-loaded liposomes). Considering the remarkable NIR-II emission property, in vivo NIR-II FLI was performed for the real time observation of NPs distribution and precise detection of tumors. As expected, the target NPs showed more intensive fluorescence signal in contrast with non-target NPs (without DSPE-PEG_2000_-GE11), which was beneficial for enhanced tumor targeting ability, by virtue of the EGFR ligand. Notably, fluorescence signals were still observed at 48 h post-injection, indicating the enormous potential of target NPs for long-term NIR-II FLI in the real-time tracing of tumors (Figure 8h). After treatment, the target NPs group provided the most significant tumor growth inhibition on both subcutaneous and orthotopic PANC-1 tumor-bearing mice models (Figure 8f,g), demonstrating that target NPs possessed extraordinary anti-tumor efficacy through synergetic type I PDT-PTT pathways. Furthermore, in all groups, no significant change of body weight of mice was discovered, as well as no noticeable changes of the complete blood panel test and serum biochemistry in the target NPs treated groups compared with the PBS treated groups, which proved the negligible systemic toxicity of the lip-DCTBT NPs. This study not only provided a paradigm for exploring advanced multifunctional type I AIE PS for the application of NIR-II FLI-guided type-I PDT-PTT synergistic therapy, but also brought favorable insights into pancreatic cancer treatment.

### 4.2. The Antimicrobial Applications

Infectious diseases caused by pathogenic microbes including bacteria, fungi, and viruses, pose serious threats to humans, since they usually cause severe diseases such as foodborne illness, tuberculosis, sepsis, meningitis, and pneumonia, for which the situation continues to worsen, along with the appearance of antibiotics-resistant microbes [71,72]. In view of this rigorous challenge, PDT has stood out as a promising candidate for antimicrobial applications including the inactivation of multidrug resistant (MDR) microbe species; ROS could provide an aggressive attack on microbes without the need for complete entrance of PSs into the microbial interior, which can potentially avoid the generation of microbial resistance [73]. In this respect, type I PDT has been widely employed due to the longer half-life of O_2_^•−^, as well as the strong oxidizing property of OH^•^. Possessing the advantage of AIE and AIG-ROS, periodical achievements in the FLI-guided PDT of pathogenic microbes have been attained, based on AIE-active type I PSs [74,75,76].

**Figure 9:**
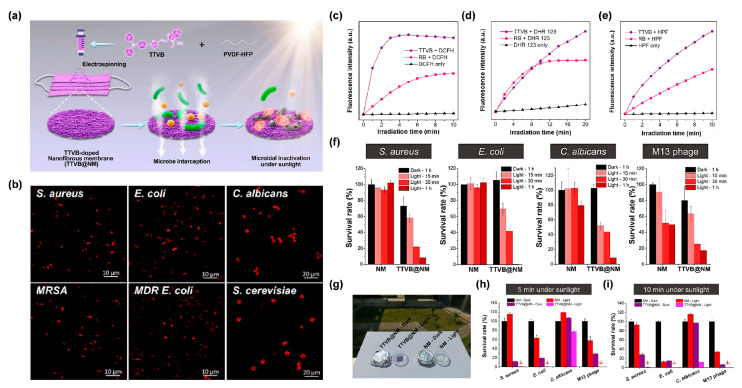
(**a**) Diagram of the preparation of TTVB@NM for antimicrobial applications. (**b**) CLSM imaging of bacteria and fungi co-incubated with TTVB. Relative fluorescence intensity of (**c**) DCFH for total ROS detection, (**d**) DHR123 for O_2_^•−^ detection, and (**e**) HPF for OH^•^ detection of TTVB and RB under light irradiation (34 mW/cm^2^). (**f**) Microbial survival rate treated with NM or TTVB@NM in dark or under sunlight irradiation. (**g**) Antimicrobial experiment against pathogenic aerosols in dark or under sunlight irradiation. Survival rate of microbes under sunlight irradiation for (**h**) 5 min and (**i**) 10 min. Reprinted with permission from [77], copyright 2021, Elsevier.

For example, Wang et al. [77] reprepared a functional nanofibrous membrane (TTVB@NM) by doping a type I AIE PSs TTVB in an electroactive polymer (PVDF-HFP) matrix using the electrospinning technique, and achieved the photodynamic elimination of pathogenic droplets and aerosols under sunlight (Figure 9a). Due to the inherent positive charge, TTVB was able to effectively stain several kinds of bacteria and fungi (Figure 9b). Under sunlight irradiation, TTVB possessed outstanding type I ROS generation efficiency (Figure 9c–e), owing to its typical D–A structure and electron-rich heteroatoms (S and N). After doping into the PVDF-HFP, the obtained nanofibrous membrane (TTVB@NM) was demonstrated to exhibit similar photophysical performances as TTVB, as well as favorable washability and photostability, indicating great potential for effective antimicrobial effect. The antimicrobial activity of TTVB@NM was subsequently verified by the significantly decreased survival rates of four kinds of pathogenic droplets (Gram-positive bacteria *S. aureus*, Gram-negative bacteria *E. coli*, fungi *C. albicans*, and M13 bacteriophage) after 1 h under sunlight irradiation (Figure 9f). Further evaluation of the antimicrobial effect of TTVB@NM against pathogenic aerosols was carried out by placing the pathogenic aerosols-loaded TTVB@NM outdoors (Figure 9g). The results revealed that TTVB@NM could effectively inactivate pathogenic aerosols containing bacteria (inhibition rate: > 99%), fungi (~88%), and viruses (>99%) within only 10 min under sunlight irradiation (Figure 9h,i). The author also stated that TTVB was measured to show moderate photothermal conversion performance, which could play an adjuvant role for microbe inhibition.

### 4.3. The Inhibition of Harmful Algal Bloom

Harmful algal bloom (HAB) has become a global environmental problem, causing serious impact on aquatic ecology and economy [78]. The rapid growth of algae aggravates O_2_ depletion and the release of harmful toxins, consequently threatening the survival of aquatic animals, resulting in widespread freshwater and marine area pollution [79]. Although many physical and chemical methods have been developed to inhibit HAB, their inherent drawbacks, such as low suppression rate, limited application area, and the possibility of secondary and persistent pollution, have hindered their widespread application [80]. In recent years, ROS-generating algaecides have aroused extensive interest owing to their effective, eco-friendly and cost-efficient properties [81]. Therefore, exploring PSs which show excellent elimination effect of algae upon light irradiation without causing toxicity to other aquatic organisms, will be a promising strategy. Of particular interest are the type I AIE PSs, which can exhibit excellent ROS generation ability under low O_2_ concentration, suitable for the relatively low O_2_ environment of algal bloom.

**Figure 10:**
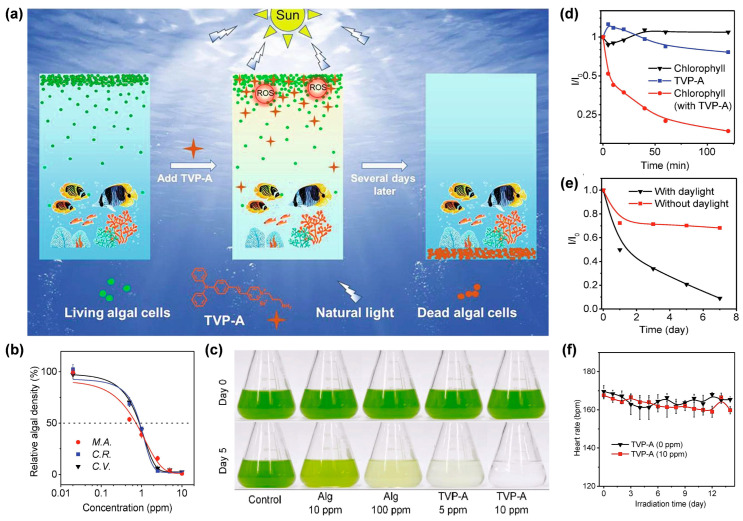
(**a**) Schematic illustration of selectively removing HAB by TVP-A upon natural light irradiation. (**b**) Relative cell density of three algae after incubating with TVP-A at different concentrations after 96 h under the simulated daily cycles. M.A.: *M. aeruginosa*; C.R.: *C. reinhardtii*; C.V.: *C. vulgaris*. (**c**) Photos of *C. reinhardtii* (1.6 × 10^7^ cells/mL) in the presence of Alg (10 ppm and 100 ppm) or TVP-A (5 ppm and 10 ppm) under the simulated daily cycles on day 0 and day 5. (**d**) The change of the relative fluorescence intensity (*I*/*I*_0_) in *C. reinhardtii* (1.6 × 10^7^ cells/mL) in the presence or absence of TVP-A (5 ppm) under different times of simulated natural light illumination. (**e**) Variation in relative absorbance of TVP-A at 462 nm with or without different natural light for evaluating the degradability of TVP-A. (**f**) The change of average heart rates of fish with or without TVP-A during the 14 days of cultivation time under the simulated daily cycles. Reprinted with permission from [82], copyright 2021, Elsevier.

Under this circumstance, Luo et al. [82] developed a water-soluble type I AIE PS with self-degrading ability, termed TVP-A, which could selectively eliminate HAB upon exposure to natural light (Figure 10a). TVP-A was constructed with a typical D–A structure with a primary amino group modified onto the terminal pyridinium, endowing the molecule with good water solubility. Moreover, the positively charged property also endowed TVP-A with a specific algae-targeting feature, on account of the negatively charged cell membrane of the algae. Upon white light irradiation, TVP-A exhibited superb ROS generation ability through both type I and type II mechanisms, particularly OH^•^. In this work, they co-incubated one cyanobacteria (*M. aeruginosa*) and two freshwater green algae (*C. vulgaris*, and *C. reinhardtii*) with TVP-A at different concentrations, respectively, under 16 h light (50 μEinstein/m^2^/s^1^)/8 h dark cycles to explore the effective concentrations in controlling the HAB. It was found that the 50% effective concentration (EC_50_) value of TVP-A was less than 1 ppm for these three kinds of algae (Figure 10b). As shown in Figure 10c, in contrast with the commercial algaecide (Alg), which still had a large amount of algae residue when the concentration was as high as 100 ppm, TVP-A exhibited ultra-efficient control of HAB, with effective inhibition of the algae bloom *C. reinhardtii* at 5 ppm and a clear color of water after five natural daily cycles at 10 ppm. The fluorescence change of chlorophyll in *C. reinhardtii* was measured to prove the irreversible damage of chloroplast due to the photodynamic process (Figure 10d). After 2 h of illumination, the fluorescence intensity of chlorophyll decreased to less than 20%, indicating that TVP-A could rapidly cause irreversible damage to these important organelles of algal cells, at especially low concentration, upon illumination. Collectively, TVP-A could be employed as a powerful agent for inhibiting HAB by destroying the chloroplast of algal cells. In addition, the strong self-degradation ability of TVP-A (Figure 10e) suggested that it was an eco-friendly agent with little environmental residue left under sufficient natural light irradiation, avoiding secondary pollution to the environment. Meanwhile, the daily heart rates of fish in the groups with or without TVP-A showed no significant difference (Figure 10f), generally indicating the good biocompatibility of TVP-A within the working concentration. This strategy afforded a favorable insight into developing novel type I ROS-generating algaecides for green HAB governance.

## 5. Conclusions and Perspectives

Profiting from the inherent advantages of AIE and AIG-ROS of AIE PSs, as well as the predominant role that type I PDT exhibited in breaking through the bottleneck of conventional type II PDT, after five years of development, AIE-active type I PS is emerging as a rising star that holds great potential in illuminating the future of PDT. Based on the recent significant advancements in novel AIE-active type I PSs, a systemic summary, involving the photophysical and photochemical mechanisms of the type I pathway, molecular design strategies, as well as practical bioapplications of type I AIE PSs, was provided in this review. In addition to a high ISC rate ensuring sufficient excited triplet-state production to undergo subsequent electron-transfer or energy-transfer pathway, approaches that could preferentially trigger the type I electron-transfer, rather than the type II energy-transfer process, are particularly needed in constructing type I PSs. Currently, according to the cascade reactions of the type I pathway, introducing functional groups with a high electron affinity to capture and stabilize the external electron, as well as enhancing the ICT intensity of PSs through donor engineering or acceptor planarization, represent two strategies to promote type I ROS generation. Guided by these strategies, a number of type I AIE PSs have been constructed and successfully applied in the type I ROS-mediated eradication of tumors and pathogen-caused infectious diseases, as well as the inhibition of harmful algae bloom. Additionally, due to the excellent features of bright fluorescence at aggregated state, rotator or vibrator-rich structure, and easy tailorability, FLI-guided type I PDT or even FLI-guided type I PDT-PTT synergistic theranostic platforms were also witnessed by ingeniously adjusting the excited-state energy dissipation pathways of type I AIE PSs.

Promising as it is, there still exist challenges or perhaps opportunities to be considered, regarding the further advance of type I AIE PSs. Primarily, although the design strategies of promoting ISC for developing highly efficient AIE PSs have already been proposed, the authoritative approach that specifically activates the following type I pathway is, to date, still far from satisfactory. In this regard, the successful examples guided by those two above-mentioned approaches are still limited, and their universal applicability remains to be validated by more attempts, despite their success in designing some type I AIE PSs. Hence, an in-depth understanding of the photochemical mechanisms of the type I pathway, the accordingly effectual design principles, as well as more ingenious trails, are urgently called for. Then, in order to avoid misjudgment about type I ROS generation capacity and to accelerate the progressive advancement of type I AIE PSs, a standard method capable of precisely assessing the quantum yield of type I ROS, particularly O_2_^•−^ and OH^•^, is highly needed. This is because current detection methods can only provide qualitative data instead of quantitative, and it is difficult to make direct comparison between the newly synthesized PSs and the previously reported ones, when only depending on qualitative data. Lastly, notwithstanding that some other bioapplications of type I AIE PSs, besides tumor treatment, have emerged, for example, the inhibition of harmful algae bloom, continuing efforts are still required for exploring a broader scope of application of type I AIE PSs, such as anti-biofilm application. We expect this review can provide valuable insights into the underlying photophysical and photochemical mechanism of the type I pathway, and inspire researchers to develop authoritative design strategies and rationales for the novel construction of ideal type I AIE PSs in the future.

## Data Availability

Not applicable. No new data were created or analyzed in this study.
